# Human RAD6 Promotes G1-S Transition and Cell Proliferation through Upregulation of Cyclin D1 Expression

**DOI:** 10.1371/journal.pone.0113727

**Published:** 2014-11-19

**Authors:** Fengfeng Cai, Ping Chen, Li Chen, Ewelina Biskup, Yan Liu, Pei-Chao Chen, Jian-Feng Chang, Wenjie Jiang, Yuanya Jing, Youwei Chen, Hui Jin, Su Chen

**Affiliations:** 1 School of Life Sciences and Technology, Department of Breast Surgery of Yangpu Hospital, Research Center for Translational Medicine at East Hospital, Tongji University, Shanghai, P. R. China; 2 Department of Oncology, University Hospital of Basel, Basel, Switzerland; 3 College of Life Sciences, Hebei United University, Tangshan, Hebei Province, P. R. China; 4 The Cancer Institute, Tangshan People’s Hospital, Tangshan, Hebei Province, P. R. China; 5 College of Life and Environmental Sciences, Wenzhou University, Wenzhou, Zhejiang Province, P. R. China; 6 Department of Biochemistry and Molecular Cell Biology, School of Medcine, Shanghai Jiao Tong University, Shanghai, P. R. China; University of Hawaii Cancer Center, United States of America

## Abstract

Protein ubiquitinylation regulates protein stability and activity. RAD6, an E2 ubiquitin-conjugating enzyme, which that has been substantially biochemically characterized, functions in a number of biologically relevant pathways, including cell cycle progression. In this study, we show that RAD6 promotes the G1-S transition and cell proliferation by regulating the expression of cyclin D1 (CCND1) in human cells. Furthermore, our data indicate that RAD6 influences the transcription of CCND1 by increasing monoubiquitinylation of histone H2B and trimethylation of H3K4 in the CCND1 promoter region. Our study presents, for the first time, an evidence for the function of RAD6 in cell cycle progression and cell proliferation in human cells, raising the possibility that RAD6 could be a new target for molecular diagnosis and prognosis in cancer therapeutics.

## Introduction

Protein ubiquitination plays multiple roles in different life processes. It is well known that protein ubiquitination is crucial for protein degradation [1]. Increasing evidence indicates that protein ubiquitination also have other biochemical functions in addition to protein degradation [2]–[4]. For example, ubiquitination of histones (e.g. H2A and H2B) always associates with gene transcriptional regulation. Multiple DNA damage repair related proteins (e.g. PCNA, FANCD2-FANCI complex etc.) can be mono- or polyubiquitinated by different E2 or E3 ubiquitin ligases. This kind of modification usually affects their abilities in the regulation of DNA damage repair and genome stability [4].

RAD6 is an E2 ubiquitin-conjugating enzyme, which exhibits its biological functions mainly through targeting different substrates for ubiquitination [5]. For instance, in *S. cerevisiae*, Rad6 participates in the regulation of DNA damage repair by catalyzing DNA clamp protein, PCNA (proliferating cell nuclear antigen), for different forms of ubiquitination. By collaborating with Rad5, Rad6 interacts with Rad18 E3 ligase to promote the monoubiquitination of PCNA at the site of lysine 164 resulting in an “Error-prone” DNA damage repair pathway. If Rad6-Rad18-Rad5 complex further interacts with UBC13 and MMS2 proteins to form a larger complex, PCNA will be polyubiquitinated at the same lysine which leads to an “Error-free” DNA damage repair pathway. Consequently, Rad6 is considered as a node for directing different pathways of DNA damage repair [6]–[8]. In addition, Rad6/HHR6 has been shown to be overexpressed in some cancer cell lines and tumors [9].

RAD6 also participates in the regulation of gene transcription through controlling H2B monoubiquitination in both mammals and *S. cerevisiae* [10]–[12]. By cooperating with its E3 ligase BRE1, RAD6 catalyzes the monoubiquitination of histone H2B at the site of lysine 120 (in mammals) or lysine 123 (in *S. cerevisae*). Besides, H2B monoubiquitination is further a determinant of two downstream histone methylations, H3K4me3 and H3K79me3 [13]–[15]. Both H2B monoubiquitination and H3K4me3 are well-known for their associations with gene activation. Both of these two histone markers play critical roles in the establishment and maintainance of an open chromatin context which is enssential for gene transcriptional activation [15], [16]. However, the function of H3K79me3 in gene transcription is relatively controversial since it has been reported that H3K79me3 is involved in both gene activation and silencing [16], [17].

Furthermore, increasing evidence is providing insights into the function of RAD6 in protein degradation [18], [19]. Our previous studies indicated that RAD6 regulates p53 protein degradation by promoting p53 ubiquitination in *Drosophila* [20]. However, it is partially different in mammals. In human cells, RAD6 regulates p53 protein levels in both transcriptional and post-transcriptional mechanisms: 1) RAD6 promotes p53 transcription by increasing the H3 methylation levels on p53 promoter region (a positive regulation, “Yang”); 2) RAD6 also promotes p53 protein degradation via ubiquitin-proteasome pathway (a negative regulation, “Yin”). This kind of “Yin-Yang” regulation mechanism of p53 protein levels by RAD6 is functionally regulated in the response to stress stimulation [21]. When cells were exposed to stress stimuli, the p53 degradation machinery composing RAD6 was disrupted, while more RAD6 were recruited to the promoter region of p53 gene locus. This kind of “Yin-Yang” mechanism allows cells to accumulate p53 protein more efficiently in response to stress stimulation [21].

Although the biochemical functions of RAD6 have been studied substantially, the biological roles of RAD6 remain to be elucidated. In this study, we demonstrate that RAD6 promotes G1-S transition and cell proliferation by regulating the expression of cyclin D1 (CCND1) which is a critical factor in the control of G1-S transition and cell proliferation. This will be the first evidence of linking RAD6 protein with cell cycle progression and cell proliferation in human cells.

## Materials and Methods

### Cell culture, plasmids and transfection

The human liver cells HL-7702 and human cervical carcinoma cells HeLa were purchased from ATCC and grown in DMEM (Gibco) containing 10% fetal bovine serum (Gibco) and antibiotics (penicillin and streptomycin) in a 5% CO_2_ incubator. Transfection of constructs into cells was performed with Lipofectamine 2000 (Invitrogen), according to the manufacturer’s standard protocol. pCMV-Myc plasmids expressing RAD6A and RAD6B were constructed by cloning RAD6A and RAD6B PCR products, which were amplified from HL-7702 cell cDNA into the pCMV-Myc vectors.

### Cell viability assay

Cell viability was analyzed by 3-(4, 5-dimethylthiazol-2-yl)-2, 5-diphenyl tetrazolium bromide (MTT) assay. 1×10^3^ cells per well were seeded in 96-well plates in five replicates. After 24 h incubation, the absorbance at 570 nm was recorded every 24 h for 5 constitutive days. 20 µL of MTT (5 mg/mL) was added into each well at each time points, and the plates were incubated at 37°C for 4 h. Following this incubation, 100 µL of dimethyl sulfoxide was added to each well to lyse the cells. The absorbance was measured using a multiwell spectrophotometer.

### RNAi knockdown of RAD6A and RAD6B

Small interfering RNAs (siRNAs) against RAD6A and RAD6B were designed and synthesized by the GenePharm Company. Transfection of siRNA into cells was performed according to the manufacturer’s protocol using Lipofectamine 2000 (Invitrogen). Briefly, 3 µg of each siRNA was transfected with 8 µL Lipofectamine 2000 per well of a 6-well plate. The sequences of the siRNAs are shown below:

siRAD6A (+):cacccucuaugaaaucaaatt,

siRAD6A (−):uuugauuucauagagggugtt;

siRAD6B (+):caguauuagcaaugaauuatt,

siRAD6B (−):uaauucauugcuaauacuggg.

### Cell cycle assay

The flow cytometric analysis was performed according to the protocol provided by the PI flow kit (KeyGEN). Briefly, the cells were stained with the PI (propidium iodide) solution for 15 min. Fluorescence was measured using a FACS Calibur apparatus (Becton Dickinson). Data collection and analysis were conducted using Cell Quest software.

### RT-PCR

A total of 4×10^6^ HL-7702 cells were lysed to extract total RNA using the TRIzol reagent (Invitrogen) according to the manufacturer’s instructions. Reverse transcription was performed as described by our previous reports [20], [21]. Total RNA (5 µg) was reverse transcribed to synthesize cDNA in a volume of 20 µL M-MLV (Moloney murine leukemia virus) reverse transcriptase (Takara). For each 25 µL PCR mixture, 1 µL of cDNA was used for 20 to 25 cycles. PCR products (10 µL) were loaded onto a 2% agarose gel, stained with ethidium bromide (EB), and photographed.

### Western blot

Cells were lysed in ATM lysis buffer (containing 100 mM Tris-Cl [pH 7.5], 150 mM NaCl, 0.2 mM EDTA, 20% glycerol, 0.4% NP-40, 2% Tween 20, and 0.2 mM PMSF). The protein concentration in the supernatant was measured with a bicinchoninic acid (BCA) assay kit (Calbiochem). SDS-PAGE was then performed using a 15% gel to resolve the proteins. After electrophoresis, proteins were transferred onto polyvinylidene difluoride (PVDF) membranes (Millipore) and hybridized with primary antibodies at the following dilutions: Myc tag (Zhongshan Golden Bridge) 1∶2000; Cyclin D1 (Santa Cruz, sc-753), RAD6 (Santa Cruz, sc-30078) and Actin (Santa Cruz, sc-69879) 1∶2000. The horseradish peroxidase (HRP)-labeled secondary antibodies (Zhongshan Golden Bridge) were all used at a dilution of 1∶4000. An ECL detection system (Amersham) was used to detect the signals on the membranes.

### Soft agar colony formation assay

For the soft agar assay, the HL-7702 cells were suspended in DMEM that contained 0.35% low melting agarose. The cells were then plated onto solidified 0.6% agarose in DMEM in 6-well culture plates at a density of 1×10^3^ cells/well. The number of the colonies was observed with a microscope (Leica, 40×) 3 weeks after seeding.

### Chromatin immunoprecipitation (ChIP) assay

ChIP assay was performed according to the published protocols of Upstate and our previous reports [20], [21].

## Results

### RAD6 regulates G1-S transition in human cells

**Figure 1:**
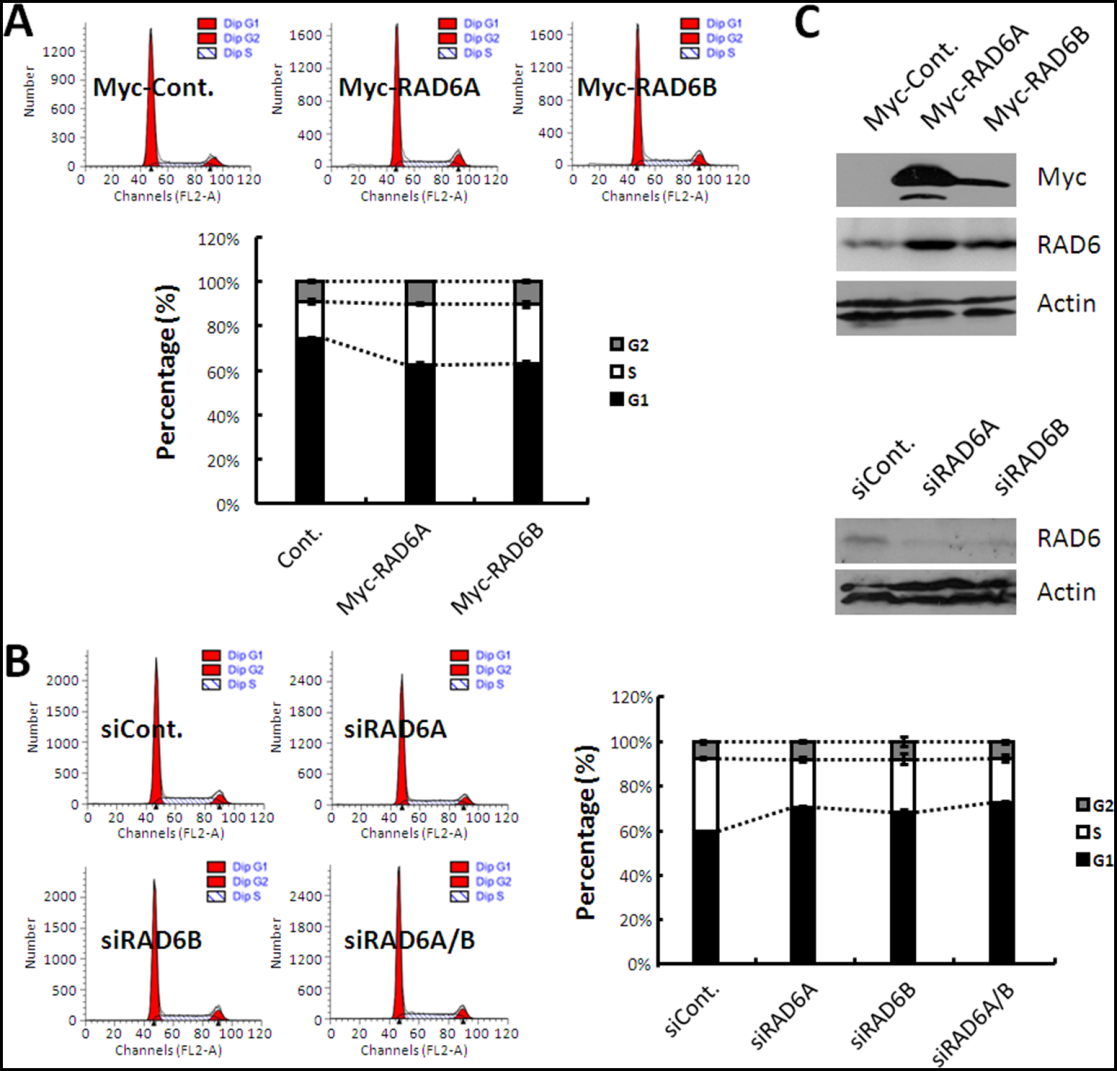
RAD6 regulates G1-S transition in human cells. (**A**) HL-7702 cells transfected with an empty control or Myc-RAD6 plasmids were stained with propidium iodide (PI) and subjected to cell cycle analysis. The quantification of the cell cycle distribution is shown below. The percentage of each phase cells was employed for the analysis of cell cycle distribution. (**B**) HL-7702 cells transfected with control or RAD6 siRNAs were stained with PI and subjected to cell cycle analysis. The quantification of the cell cycle distribution is shown on the right. The percentage of each phase cells was employed for the analysis of cell cycle distribution. (**C**) The expression levels of RAD6 in cells used in the above cell cycle assays (overexpression, upper; knockdown, lower) were analyzed by western blot assay.

To determine the biological functions of RAD6 in human cells and its potential effects on tumorigenesis, we first tested the effect of RAD6 on cell cycle progression. HL-7702 cells transfected with Myc-Control (Cont.), Myc-RAD6A or Myc-RAD6B plasmids were harvested and subjected to cell cycle assay. As shown in **Figure 1A**, RAD6 overexpression resulted in an increase of S phase cells (Cont.: 17%, RAD6A: 27% and RAD6B: 26%) and a decrease of G1 phase cells (Cont.: 74%, RAD6A: 62% and RAD6B: 63%). However, we observed no obvious change of the population of G2/M phase cells. This result indicates that overexpression of RAD6 promotes G1-S transition in HL-7702 cells.

We also determined whether the knockdown of RAD6 expression would result in an opposite effect on the cell cycle progression. We performed similar cell cycle assay by using cells transfected with control (cont.) or RAD6 siRNAs (RAD6Ai and RAD6Bi). As we expected, knockdown of RAD6 expression by RNAi results in the decrease of S phase cells (Cont.: 33%, RAD6Ai: 22%, RAD6Bi: 24% and RAD6A/Bi: 20%) and increase of G1 phase cells (Cont.: 60%, RAD6Ai: 71%, RAD6Bi: 68% and RAD6A/Bi: 72%). There is also no obvious change of the G2/M cell population (**Figure 1B**). Additionally, we determined the RAD6 expressional levels of the cells used in our cell cycle assay (**Figure 1C**). We repeated all of the above experiments in HEK293T and HeLa cells, and similar results were obtained (**Figure S1** and **Figure S2B**). Taken together, our results suggest that RAD6 regulates the G1-S transition in human cells.

### RAD6 regulates cell proliferation in human cells

**Figure 2:**
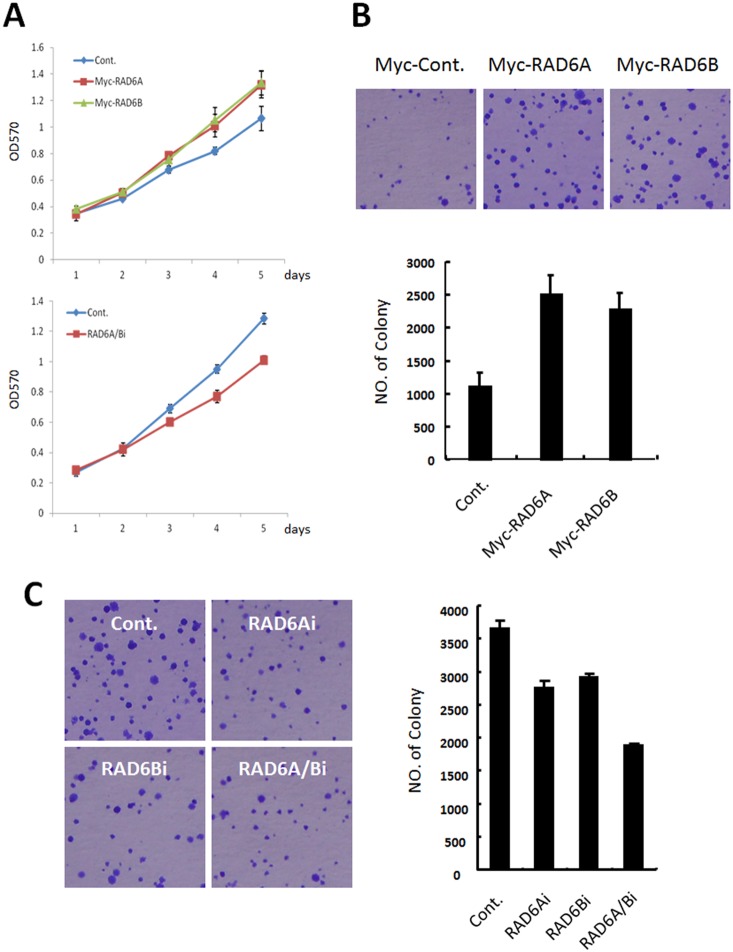
RAD6 regulates cell proliferation and tumor growth in vitro. (**A**) HL-7702 cells transfected with an empty control or Myc-RAD6 plasmids, or a control siRNA or RAD6 specific siRNAs were subjected to MTT assay. OD570 value was examined for the analysis of cell proliferation. Five replicates of each treatment were employed in this assay (n = 5). (**B**) HL-7702 cells transfected with an empty control or Myc-RAD6 plasmids were subjected to soft agar colony formation assay. The quantification of the colony number is shown below. Three replicates of each treatmen were employed in this assay (n = 3). (**C**) HL-7702 cells transfected with control or RAD6 shRNAs were subjected to soft agar colony formation assay. The quantification of the colony number is shown on the right. Three replicates of each treatmen were employed in this assay (n = 3).

Since we observed that RAD6 promotes G1-S transition which has been recognized as an important characteristic for elevated cell proliferation in human cells (**Figure 1**), we next investigated whether RAD6 plays a role in regulation of cell proliferation. HL-7702 cells were transfected with either an empty vector (pCMV-Myc, Cont.), Myc-tagged RAD6-expressing vectors (Myc-RAD6A and Myc-RAD6B), or a siRNA control (Cont.), RAD6 specific siRNAs. MTT assay was performed and showed that overexpression of RAD6 stimulates cell proliferation, while knockdown of RAD6 expression inhibits cell proliferation (**Figure 2A**). Colony formation capability is another critical characteristic indicating cell proliferation and tumor growth activity *in vitro*. Therefore, Control (Cont.) and Myc-RAD6 transfecting HL-7702 cells were subjected to soft agar colony formation assay. As shown in **Figure 2B**, we observed that the colony number and size increased in RAD6 overexpressing cells compared with control cells. In contrast, knockdown of RAD6 decreased the colony formation capacity of HL-7702 cells (**Figure 2C**). We also obtained similar results in HeLa cells (data not shown).

Taken together, these results suggest that RAD6 regulates cell proliferation and tumor growth activities of human cells *in vitro*, which is consistent with the effect of RAD6 on the cell cycle progression (**Figure 1**).

### RAD6 regulates the expression of cyclin D1 (CCND1)

**Figure 3:**
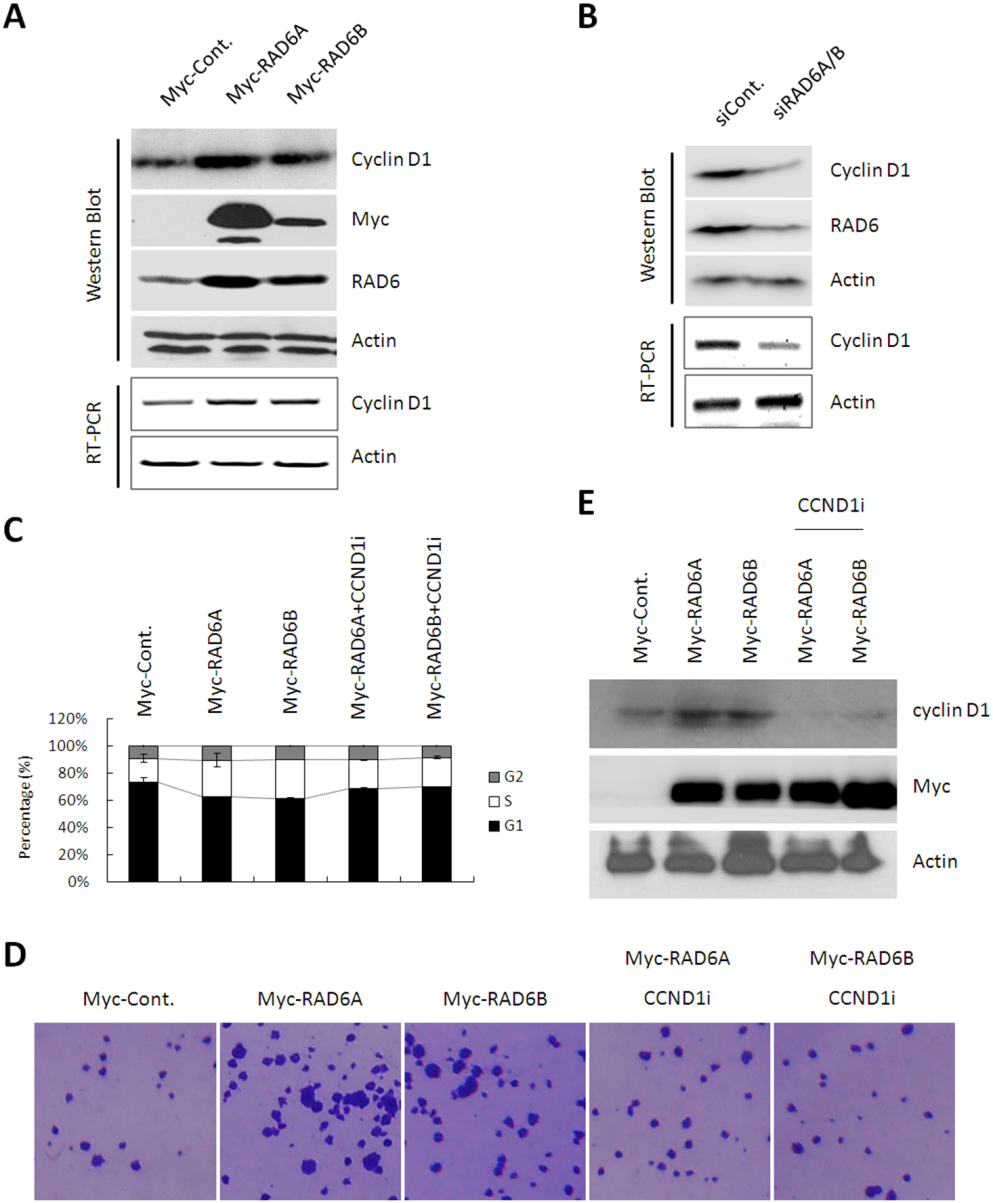
RAD6 regulates the expression of cyclin D1 in both the mRNA and protein level. (**A**) HL-7702 cells transfected with an empty control or Myc-RAD6 expressing plasmids were lysed and subjected to RT-PCR and western blot assays. (**B**) HL-7702 cells transfected with control or RAD6 siRNAs were lysed and subjected to RT-PCR and western blot assays. (**C**) HL-7702 cells transfected with an empty control and Myc-RAD6 plasmids together with or without CCND1 siRNA were harvested and stained with PI, and then cells were subjected to cell cycle assay. The quantification of the cell cycle assay is shown. (**D**) Soft agar colony formation assays were performed using control, or RAD6 overexpressing, or RAD6 overexpressing together with CCND1 knocking down HL-7702 cells. (**E**) HL-7702 cells used in the above assays were lysed and subjected to western blot analyses with antibodies against CCND1, Myc-tag and Actin as indicated.

It is well known that cyclin D1 (CCND1) plays a critical role in the regulation of G1-S transition and cell proliferation. CCND1 forms complex with its specific kinase, CDK4 or CDK6, in early G1 phase, and these complexes functions during the entire G1 to S phase transition [22]–[24]. The CCND1-CDK4 or CCND1-CDK6 kinase complex can phosphorylate the retinoblastoma (RB) protein, and then activates the E2F transcription factor enventually leading to the accomplishment of G1-S transition [24]. Since the effects of RAD6 that we observed on cell cycle progression and cell proliferation are positively correlated with those of CCND1. Therefore, we next tested whether the expression of CCND1 is affected by RAD6. HL-7702 cells were transfected with control (Cont.) or Myc-RAD6 plasmids (RAD6A and RAD6B). After 48 h of transfection, cells were lysed and subjected to RT-PCR and western blot assays. As expected in our hypothesis, both the mRNA and protein levels of CCND1 were upregulated in RAD6 overexpressing cells (**Figure 3A**). In addition, the knockdown experiment was performed with RAD6 siRNAs. Accordingly, an opposite result was obtained: knockdown of RAD6 decreased the mRNA and protein levels of CCND1 in HL-7702 cells (**Figure 3B**). We also repeated the above experiments in HeLa cells, and similar results were obtained (**Figure S2A**).

To determine whether other cyclins can also be affected by RAD6, we examined the protein levels of Cyclin A, Cyclin E, Cyclin B1 and Chk2 in control and RAD6 overexpressing HL-7702 cells. However, all these examined proteins did not show obvious changes in response to RAD6 overexpression (**Figure S3**) suggesting CCND1 would probably be the only affected cyclin upon RAD6 alteration.

These results demonstrate that RAD6 regulates the expressional levels of CCND1, which probably contributes to the effect of RAD6 on cell cycle progression and cell proliferation.

### RAD6 induced G1-S transition and cell proliferation is mediated by CCND1

To determine whether RAD6 induced G1-S transition and cell proliferation are mediated by the upregulation of CCND1 expression, we performed a rescue assay by knocking down the expression of CCND1. HL-7702 cells transfected with empty control (Cont.), Myc-RAD6 together with or without CCND1 siRNA were harvested and subjected to cell cycle assay. Our results show that G1-S transition promoted by RAD6 overexpression was rescued by CCND1 knockdown (**Figure 3C**). As expected, RAD6 overexpression induced enhancement of cell proliferation was also partially inhibited by CCND1 knockdown (**Figure 3D**). The expressional levels of the related proteins in the cells used in the above assays were also determined (**Figure 3E**). We repeated the above experiments in HeLa cells, and similar results were obtained (**Figure S2B and C**).

Taken together, our results suggest that RAD6 induced G1-S transition and cell proliferation is indeed mediated by the upregulation of CCND1 expression.

### RAD6 regulates CCND1 transcription via modulating the levels of H2B monoubiquitination and H3K4me3 on the CCND1 promoter region

**Figure 4:**
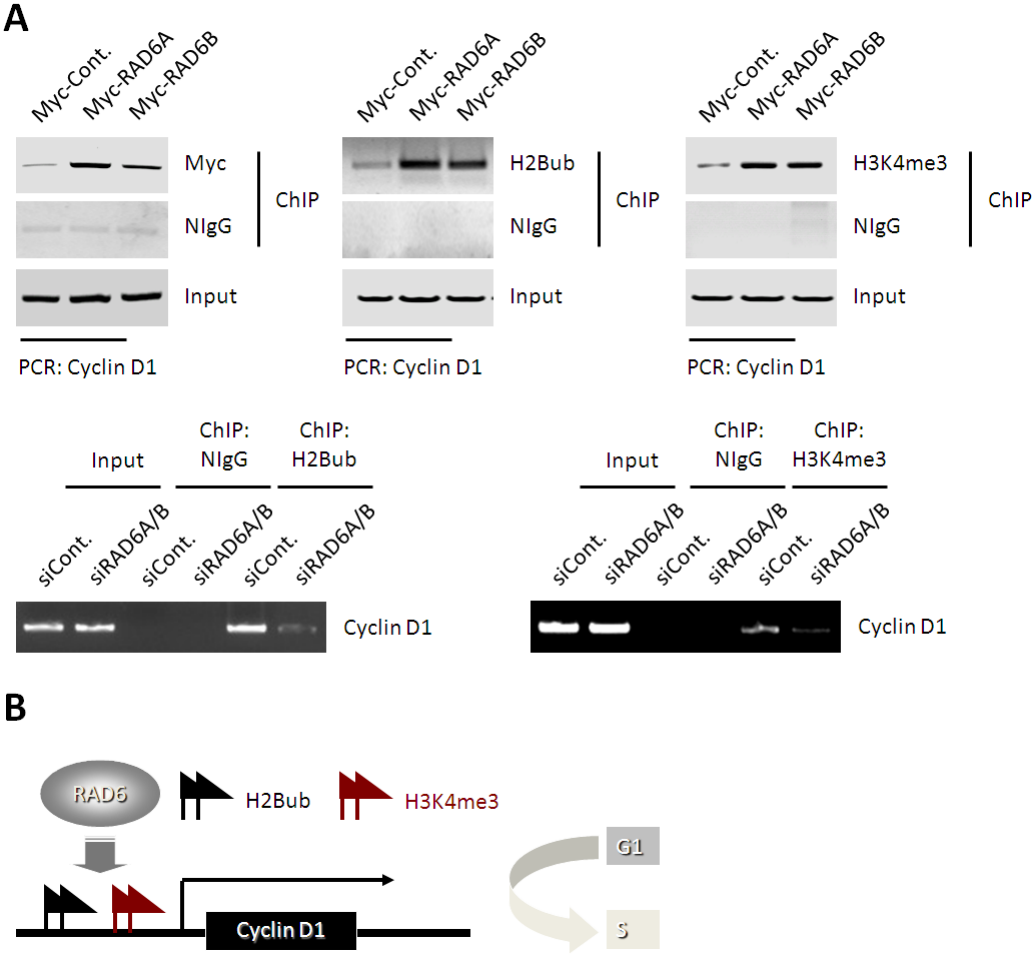
RAD6 enhances the enrichment of H2B monoubiquitination and H3K4me3 level at CCND1 promoter region. (**A**) HL-7702 cells transfected with an empty control or Myc-RAD6 expressing plasmids, or a control siRNA or RAD6 specific siRNAs were lysed and sonicated, then subjected to chromatin immunoprecipitation (ChIP) assays with antibodies against Myc-tag (indicating exogenous RAD6), H2B monoubiquitination and H3K4me3. Nonspecific antibody, normal rabbit IgG (NIgG), was used as a negative control. The precipitated DNA fragments were supplied for PCR assay with primers specific for CCND1 promoter. (**B**) Working model. RAD6 regulates the transcription of CCND1 likely through affecting the H2B monoubiquitination and H3K4me3 levels at the CCND1 promoter region.

H2B monoubiquitination and H3K4me3 are two well-known histone markers for gene activation, and both of them are believed to be the downstream targets of RAD6. The above results have shown that the CCND1 mRNA levels are positively correlated with the RAD6 protein levels (**Figure 3**). We therefore examined whether RAD6 regulates CCND1 transcription directly by modulating the H2B monoubiquitination and H3K4me3 levels at the CCND1 promoter region. HL-7702 cells were transfected with an empty control (Cont.) or Myc-RAD6 plasmids (Myc-RAD6A and Myc-RAD6B). Then, chromatin immunoprecipatation (ChIP) assays were performed with antibodies against Myc-tag, H2B monoubiquitination and H3K4me3. We observed that RAD6 directly binds on the *CCND1* promoter region (**Figure 4A** **, upper left**), and the levels of H2B monoubiquitination and H3K4me3 are increased in RAD6 overexpressing cells compared with the control cells (**Figure 4A** **, upper middle and right**). We also examined the effect of RAD6 RNAi on the enrichments of H2B monoubiquitination and H3K4me3 on *CCND1* promoter, and an opposite result to RAD6 overexpression were observed supporting the same conclusion of RAD6 on *CCND1* expression (**Figure 4A** **, lower**).

Taken together, these results suggest that RAD6 might regulate the transcription of CCND1 through modulating the H2B monoubiquitination and H3K4me3 levels at the *CCND1* promoter region (**Figure 4B**).

## Discussion

G1-S transition is critical for cell proliferation and tumor growth [22]. Therefore, understanding the regulation mechanism and finding novel regulator of G1-S transition is essential for the development of cancer molecular diagnosis and anticancer therapeutics.

In this study, we demonstrated for the first time that RAD6 is a novel regulator of G1-S transition and cell proliferation in human cells. Our results suggest that RAD6 overexpression promotes G1-S transition and cell proliferation, while knockdown of RAD6 expression inhibits G1-S transition and cell proliferation (**Figure 1** and **Figure 2**).

CCND1 has been recognized as one of the most important regulators for G1-S transition [23], [24]. By cooperating with its specific kinase, CDK4 or CDK6, CCND1 promotes the phosphorylation of RB, and activates the transcription factor E2F, and is enssential for G1-S transition. CCND1-CDK4 or CCND1-CDK6 complex play roles during the whole G1-S transition process, while other cyclins (e.g. Cyclin E) play limited time periods [22]–[24]. We therefore tested the effect of RAD6 on CCND1 expression. Indeed, RAD6 overexpression upregulates the expression of CCND1 at both the mRNA and the protein levels, whereas knockdown of RAD6 expression results in an opposite effect. These data suggest that RAD6 is a regulator of CCND1 expression. Furthermore, we aimed to determine the potential molecular mechanism by which RAD6 regulates the expression of CCND1. Results of our chromatin IP experiment showed that RAD6 is enriched at the CCND1 promoter region, and the H2B monoubiquitination and H3K4me3 levels are also increased in RAD6 overexpressing HL-7702 cells (**Figure 4A**). These data are consistent with the previous reports, where RAD6 has been described as the upstream regulator of H2B monoubiquitination and H3K4me3, and both of these two histone modifications are involved in gene transcriptional activation [10], [13]–[17]. For instance, monoubiquitination of H2B can loosen the nucleosome leading to an opened chromatin structure allowing for the access of transcription activation related factors [16]. Therefore, for the first time here, our results indicate that RAD6 promotes the expression of CCND1 through upregulation of the H2B monoubiquitination and H3K4me3 levels at the *CCND1* promoter (**Figure 4B**), and further contributes to the cell cycle progression and cell proliferation.

It is worth mentioning that our results are partially consistent with a relative new report, in which *S. Cerevisiae* model system has been used. They observed that Rad6-Bre1 complex modulates the transcription of many cyclin genes (including CLN2 and CLB5) through directly binding on the regulatory regions of the related cyclin genes, and further promotes the cell cycle entry in *S. Cerevisiae* [25]. Both CLN2 and CLB5 are G1 phase cyclins functioning in promoting G1 to S progression in yeast. We also examined the responses of some other cyclins (including Cyclin A, Cyclin E, and Cyclin B1) to RAD6 overexpression. Unfortunately, among them, CCND1 is the only altered protein (**Figure S3**) suggesting that there could still be some differences between human and yeast about the roles of RAD6 in regulating the expression of cell cycle related genes.

Here, our work indicates that RAD6 is a novel regulator of G1-S transition and cell proliferation in human cells by targeting CCND1 expression. As previously mentioned, both CCND1 and its subsequent G1-S transition are significantly involved in tumorigenesis. Therefore, these findings further raised the possibility that RAD6 could be a new target for molecular diagnosis and prognosis in cancer therapeutics.

## Supporting Information

Figure S1 **Knockdown of RAD6 inhibits G1-S transition in HEK293T cells.** HEK293T cells transfected with a control siRNA or RAD6 specific siRNAs were used for cell cycle assay. The quantification of the cell cycle distribution is shown right. The percentage of each phase cells was employed for the analysis of cell cycle distribution.
(TIF)

Figure S2 **RAD6 regulates the expression of cyclin D1 in both the mRNA and protein level in HeLa cells.** (**A**) HeLa cells transfected with an empty control or Myc-RAD6 expressing plasmids, or a control siRNA or RAD6 specific siRNAs were lysed and subjected to RT-PCR and western blot assays. (**B**) HeLa cells transfected with an empty control and Myc-RAD6 plasmids together with or without CCND1 siRNA were harvested and stained with PI, and then cells were subjected to cell cycle assay. The quantification of the cell cycle assay is shown. (**C**) Soft agar colony formation assays were performed using control, or RAD6 overexpressing, or RAD6 overexpressing together with CCND1 knocking down HeLa cells.
(TIF)

Figure S3 **RAD6 overexpression does not affect the protein levels of cyclin A, cyclin E, cyclin B1 and Chk2.** HL-7702 cells transfected with an empty control or Myc-RAD6 expressing plasmids were lysed and subjected to western blot assays with antibodies as indicated.
(TIF)
