# Supplementary figures and images for: Human RAD6 Promotes G1-S Transition and Cell Proliferation through Upregulation of Cyclin D1 Expression
Source: PLoS One. 2014 Nov 19;9(11):e113727. doi: 10.1371/journal.pone.0113727 (PMC4237501; doi:10.1371/journal.pone.0113727)

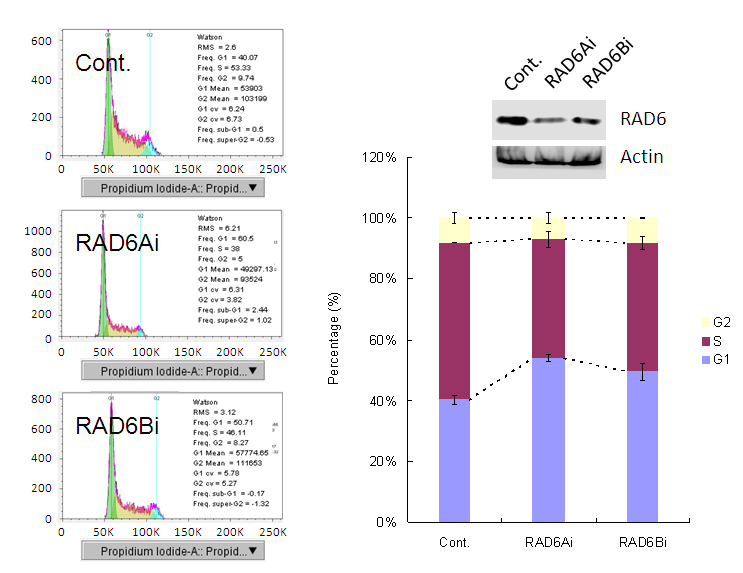

Supplement: Figure S1 — Knockdown of RAD6 inhibits G1-S transition in HEK293T cells. HEK293T cells transfected with a control siRNA or RAD6 specific siRNAs were used for cell cycle assay. The quantification of the cell cycle distribution is shown right. The percentage of each phase cells was employed for the analysis of cell cycle distribution. (TIF) [file pone.0113727.s001.tif]

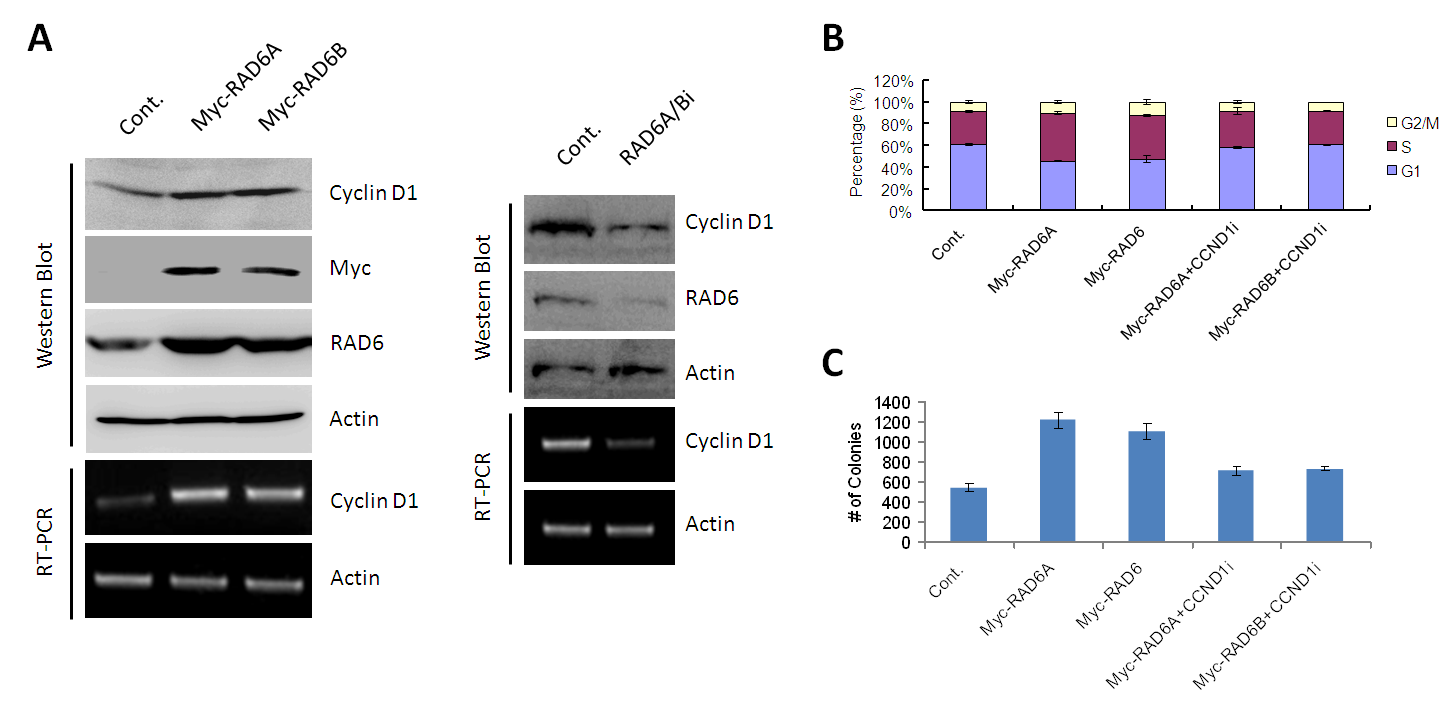

Supplement: Figure S2 — RAD6 regulates the expression of cyclin D1 in both the mRNA and protein level in HeLa cells. (A) HeLa cells transfected with an empty control or Myc-RAD6 expressing plasmids, or a control siRNA or RAD6 specific siRNAs were lysed and subjected to RT-PCR and western blot assays. (B) HeLa cells transfected with an empty control and Myc-RAD6 plasmids together with or without CCND1 siRNA were harvested and stained with PI, and then cells were subjected to cell cycle assay. The quantification of the cell cycle assay is shown. (C) Soft agar colony formation assays were performed using control, or RAD6 overexpressing, or RAD6 overexpressing together with CCND1 knocking down HeLa cells. (TIF) [file pone.0113727.s002.tif]

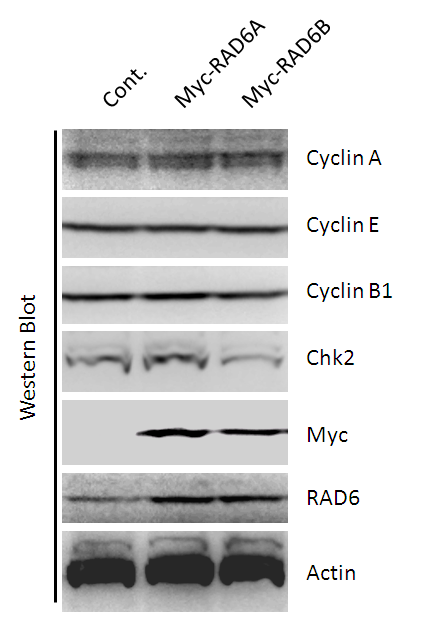

Supplement: Figure S3 — RAD6 overexpression does not affect the protein levels of cyclin A, cyclin E, cyclin B1 and Chk2. HL-7702 cells transfected with an empty control or Myc-RAD6 expressing plasmids were lysed and subjected to western blot assays with antibodies as indicated. (TIF) [file pone.0113727.s003.tif]
